# Correlation between depression, anxiety, and polymorphonuclear cells’ resilience in ulcerative colitis: the mediating role of heat shock protein 70

**DOI:** 10.1186/1471-230X-14-77

**Published:** 2014-04-17

**Authors:** Ilias I Vlachos, Calypso Barbatis, Maria Tsopanomichalou, Lydia Abou-Assabeh, Konstantinos Goumas, Maria Ginieri-Coccossis, Marina Economou, George N Papadimitriou, Efstratios Patsouris, Polyxeni Nicolopoulou-Stamati

**Affiliations:** 1 First Department of Pathology, Medical School, Athens University, Athens, Greece; 2 Pathology Department, Hellenic Red Cross Hospital, Athens, Greece; 3 Gastroenterology Department, Hellenic Red Cross Hospital, Athens, Greece; 4 1st Department of Psychiatry, University of Athens, Eginition Hospital, Athens, Greeece; 5 First Department of Pathology, Laiko Hospital, Athens University, Athens, Greece; 6 Ilias Vlachos, 8 str Alkmanos, 11528 Athens, Greece

**Keywords:** Ulcerative colitis, Polymorphonuclear cells, Heat shock protein 70, Anxiety, Depression, Psychoneuroimmunology

## Abstract

**Background:**

To investigate whether anxiety and depression levels are associated with Heat Shock Protein 70 (HSP70) induction in the colon of patients with ulcerative colitis (UC).

**Methods:**

The design was cross-sectional. Clinical activity was assessed by the Rachmilewitz Index (CAI). Three psychometric questionnaires were used: Zung Depression Rating Scale (ZDRS), Spielberg State-Trait Anxiety Inventory (STAI), Hospital Anxiety and Depression Scale (HADS). Colon biopsies were obtained from each affected anatomical site. Severity of inflammation was assessed by eosin/hematoxylin. Constitutive (HSP70c) and inducible (HSP70i) HSP70 expression were immunohistochemically studied.

**Results:**

29 UC patients were enrolled (69% men). Mean age was 46.5 years (SD: 19.5). Inflammation severity was moderate in 17 patients, severe in 6, and mild in 6. The mean number of years since diagnosis was 7.9 (SD: 6.5). The mean CAI was 6.4 (SD: 3.1). In active UC, there was downregulation of HSP70c in inflamed epithelium, without significant HSP70 induction. In 22/29 cases of active cryptitis, polymorphonuclear cells (PMN) clearly expressed HSP70i, with weak, focal positivity in the other 7 cases. Except for the hospital anxiety scale, scores in all psychometric tools were higher in patients with strong HSP70i immunoreactivity in the PMN. Logistic regression showed a strong positive relationship between HSP70i immunoreactivity in the PMN cells and scores in the trait anxiety, ZDRS, and hospital depression scales, (Odds ratios 1.3, 1.3, and 1.5; P = 0.018, 0.023, and 0.038; Wald test, 5.6, 5.2, and 4.3 respectively) and a weaker but significant positive correlation with the CAI (Odds ratio 1.654; P = 0.049; Wald test 3.858).

**Conclusion:**

HSP70 is induced in PMN cells of UC patients and its induction correlates with depression and anxiety levels.

## Background

Ulcerative colitis (UC) represents one of the major idiopathic inflammatory bowel diseases (IBD), along with Crohn’s disease (CD). Although its etiology remains unknown, there is evidence of an aberrant response of the immune system to commensal microbial flora in a genetically susceptible host [1].

Psychological stress and depression are known to prolong the clinical course of UC in terms of symptom severity and relapses [2-4] and it seems that UC patients, compared to the general population, are significantly more likely to have a diagnosis of anxiety and major depression [5,6]. Animal research depicts a causal relationship between experimentally induced depression and increased secretion of proinflammatory cytokines leading to reactivation of colitis. Τricyclic antidepressants and selective serotonine reuptake inhibitors seem to attenuate the inflammatory effect of proinflammatory cytokines [7,8] as well as to exert analgesia on IBD patients [9]. Moreover, UC patients seem to have an increased sympathetic autonomic activity compared to controls, and the severity of their symptoms is associated with measures of personality-related but not situational anxiety [10]. Mawdsley and colleagues showed that acute psychological stress induces inflammatory responses in UC patients [3]. UC patients seem to be influenced more than CD patients by external factors and to a lesser extent by genetic factors. Identical twin studies reveal smaller genetic predisposition for the etiology of UC (10%), compared to the 50% genetic predisposition of identical twins with CD [11]. In this light, the theory of neuroimmunomodulation in UC requires further documentations [12,13].

Heat shock proteins (HSPs) are ubiquitous in all living organisms and cells and form the most virulent cellular defense for a variety of stressors that disrupt cell proteins and threaten cell survival. Types of cellular stress, proven to promote Hsp induction, are: thermal stress -as stated in their etymology-, oxidative stress through the formation of reactive oxygen species (ROS), which disturb the cell by oxidizing lipids of the membrane, its proteins and even the cellular DNA leading the cell to apoptosis or cell death. Other types of cellular stress are bacteria and bacterial exo-and endotoxins, viral infections, cytokines, ischemia. Psychophysiological stress has also been associated with HSP70 induction, mainly in animal models [13,14].

They exist in two forms: the constitutive and the inducible. The inducible form becomes activated under conditions of cellular stress and exerts cytoprotective functions [12,13]. Heat shock proteins act as molecular chaperones by rescuing essential cell proteins and preventing aggregation of denatured ones [15] and they inhibit cellular apoptosis by suppressing parts of the apoptotic machinery [16]. Animal studies have shown that HSPs are also induced in different tissues via the hypothalamic-pituitary-adrenal (HPA) axis and the sympatho-adrenomedullary system (SAS) under conditions of psychophysiological stress [17,18].

The inducible HSP70 (Hsp70i), along with the inducible HSP25/27, is associated more with the protection of the intestinal mucosa compared to other members of the heat shock protein family. Its downregulation in IBD, constitutes a potentially dangerous situation, since the intestinal mucosa becomes susceptible to immune and inflammatory processes [19-21].

Its induction in UC remains a topic of dispute, as relevant publications show evidence of either enhanced expression or downregulation [19,22]. Psychosomatic research stresses the need for further exploration in humans of the role that HSPs play as possible mediators between gut inflammation and susceptibility to psychological stress [23].

In the present study, we investigated whether anxiety and depression levels evaluated by relevant psychometric tools are associated with HSP70 induction in the colon of patients with UC, as a reflection of the emotional state on a histological level.

## Methods

This was a cross-sectional study. Participants were recruited from patients hospitalized for coloscopic investigation for possible relapse of their existing IBD. The recruitment took place in the gastroenterology department of the tertiary care “Hellenic Red Cross Hospital” in Athens, between October 2008 and June 2010.

The diagnosis of UC was made following the standard clinical, radiological, and histopathological procedures described by the Lockhart-Mummery and Morson criteria [24]. Patients with CD were excluded, because the different nature and progress of the diseases (UC vs. CD) has proven to lead to Type II statistical errors (false negative results), when samples of patients are mixed according to relevant research [23].

Eligible patients were informed about the study aim and procedures and those who agreed to participate and signed informed consent were enrolled into the study. The study was conducted according to the Declaration of Helsinki and was approved by the General Assembly of the medical School of the University of Athens with the protocol number 3049/1.12.03.

### Study procedures

Disease activity was assessed clinically with the Rachmilewitz Colitis Activity Index (CAI) [11]. CAI includes the evaluation by a gastroenterologist of seven domains that imply disease activity: number of stools weekly, blood in stools, investigator’s global assessment of symptomatic rate, abdominal pain or cramps, temperature due to colitis, extraintestinal manifestations, and laboratory findings focused on sedimentation rate and hemoglobin. A CAI ≥ 6 is considered indicative of active disease. Disease duration, age, and gender were also recorded.

The patients completed three psychometric questionnaires: the Zung Depression Rating Scale (ZDRS), the Spielberg State-Trait Anxiety Inventory (STAI) Form X I, II as state and as trait, and the Hospital Anxiety and Depression Scale (HADS).

In addition, intestinal biopsies were taken and were diagnosed blinded by two pathologists. The type and severity of inflammation were assessed on each section with hematoxylin & eosin staining. The site and intensity of expression of HSP70 expression were studied immunohistochemically.

### Psychometric tools

We used the Greek standardized versions of three self-reported instruments frequently used in research concerning anxiety and depression in IBD patients [4]:

The STAI form consists of two 20-items questionnaires [25,26]. The first questionnaire measures state anxiety, i.e. how the respondent “feels right now” meaning the time of completion. The second questionnaire measures trait anxiety, i.e. how the respondent generally feels. For each questionnaire, the scores range is 20–80. The cut-point for clinically significant anxiety is 39–40, scores > 54 are considered indicative of a mental disorder [27].

The ZDRS [28,29] is a self-rating scale for the measurement of depression. It consists of 20 items that cover affective, psychological, and somatic symptoms. The respondent specifies the frequency with which the symptom is experienced (from 1 = little to 4 = most of the time) for the past several days. The minimum scores is 20 and the maximum score is 80. Scores > 50 indicate clinical depression.

The HADS [30,31] was developed to identify possible and probable cases of anxiety and depression among patients in non-psychiatry hospital clinics and has been extensively validated in chronic diseases including IBD [32]. The HADS consists of two subscales: the HA for anxiety, and the HD for depression. The minimum scores is 0 and the maximum score is 21. Scores >7 indicate “possible case”, and > 11 indicate “probable case”. The questionnaire asks how the respondent has been feeling during the past week.

### Biopsies

Colonic biopsies were obtained from each anatomical site of the colon, during coloscopy. As control for the HSP70 monoclonal antibody, biopsies from the normal xcolon of patients with adenocarcinoma (resection margins with no presence of active inflammation, no polymorphonuclear cells, no histological abnormalities and more than 20 cm distance from the tumour), were used after informed consent. No psychometric questionnaires were given to adenocarcinoma patients.

The tissue was fixed in neutral buffered formalin. Sections were stained with hematoxylin & eosin. The severity of active disease was semiquantitatively assessed using an accepted scoring system ranging from 0–3 as well as the distinction between actively inflamed from uninvolved tissues, using a scoring system from 0 to 3 (0 = no activity, 1 = mild, 2 = moderate, 3 = severe) [33,34].

Constitutive and inducible forms of the HSP70 protein were detected immunohistochemically on 3 μm thick sections with the Bond-MAX system, Leica Ltd. The following mouse monoclonal antibodies were used: anti-HSP70 Ad-2 (clone W27, Lab vision Corp USA, diluted 1:50), anti-HSP70i (SPA-810, clone C92F3A-5, Stressgen, USA, diluted 1:25). Both antibodies are specific for mammalian HSP70 and do not crossreact with bacterial antigens. SPA-810 has been validated in previous methodological and clinical studies on HSP70 induction [22,35]. HSP70 expression (both constitutive and inducible) was assessed in each separate biopsy specimen regarding: surface epithelium (nuclear and cytoplasmic), crypts (nuclear and cytoplasmic), lymphoid tissue, monocytes (MC), and neutrophils (PMN), using an accepted system marking: absence as “0”, low staining as “+/-“, moderate as “+”, and intense staining as “++” [22,35].

### Statistical analysis

Descriptive statistics were used for demographic and clinical characteristics. Based on the psychometric scale cut-offs, we distinguished between three levels for anxiety and depression: normal, significant case, and clinical case. There was a small number of missing values (4 missing points in the ZDRS measurements) which were considered as missing completely at random (MCAR). Pearson chi-square was used for the comparison of immunoreactivity levels by inflammatory activity. t-tests for independent samples, and Mann–Whitney test was used for comparisons of means in the scales used, between the group of patients with positive or negative staining in HSP70i. Logistic regression was used to determine the effect of psychometric scores on HSP70i immunoreactivity. Although we used the four point scale [22,35] for the surface epithelium the crypts and the lamina propria, in PMN cells our attention was driven to those cells that didn’t show any HSP70 induction, despite active disease. Therefore, in the statistical analysis we used a dichotomous scale between present or absent HSP70 induction in PMN cells of active ulcerative colitis patients. PMN cells were not classified according to the intensity of the staining but on the basis of positivity or negativity. Moreover, positive cells were stained (by SPA-810 antigen), while negative cells were not stained.

## Results

29 patients with UC were enrolled, 20 men (69%) and 9 women (31%). The mean age was 46.5 years (SD: 19.5). Disease activity was mostly moderate (58.6% of patients); severe disease was present in 20.7% and mild disease in 20.7%. The mean number of years since diagnosis was 7.9 (SD: 6.5) for the 25/29 patients, because 4/29 patients were not sure about the specific year of diagnosis. 10 patients (35%) were receiving corticosteroids. The mean CAI was 6.4 (SD: 3.1).

### Immunohistochemistry - controls

**Figure 1:**
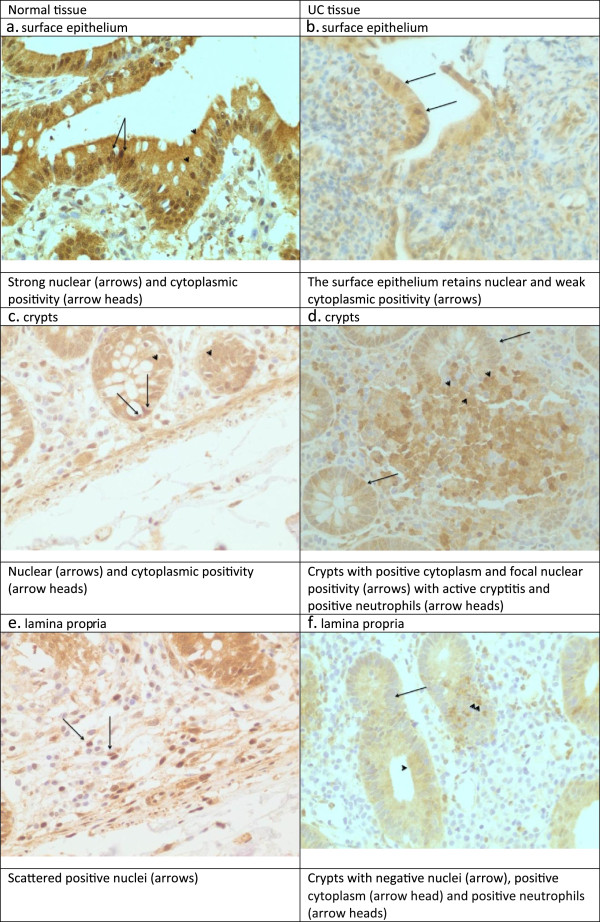
**Expression of constitutive HSP70 in the epithelium, crypts, and lamina propria of normal and UC tissue. a**. Normal tissue. Surface epithelium. Strong nuclear (arrows) and cytoplasmic positivity (arrow heads). **b**. UC tissue. Surface epithelium. The surface epithelium retains nuclear and weak cytoplasmic positivity (arrows). **c**. Normal tissue. Crypts. Nuclear (arrows) and cytoplasmic positivity (arrow heads). **d**. UC tissue. Crypts. Crypts with positive cytoplasm and focal nuclear positivity (arrows) with active cryptitis and positive neutrophils (arrow heads). **e**. Normal tissue. Lamina propria. Scattered positive nuclei (arrows). **f**. UC tissue. Lamina propria. Crypts with negative nuclei (arrow), positive cytoplasm (arrow head) and positive neutrophils (arrow heads).

For the constitutive HSP70, we found strong nuclear and cytoplasmic expression throughout the epithelium with the same intensity from the surface to the base of the crypts and the MC of the lamina propria (Figure 1a, c, e).

**Figure 2:**
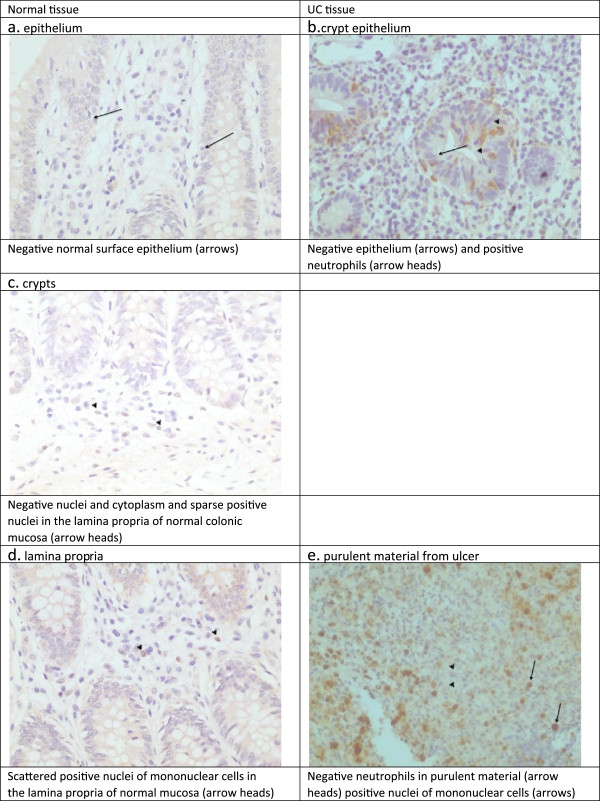
**Expression of inducible HSP70 in the epithelium, crypts, and lamina propria of normal and UC tissue. a**. Normal tissue. Epithelium. Negative normal surface epithelium (arrows). **b**. UC tissue. Crypt epithelium. Negative epithelium (arrows) and positive neutrophils (arrow heads). **c**. Normal tissue. Crypts.Negative nuclei and cytoplasm and sparse positive nuclei in the lamina propria of normal colonic mucosa (arrow heads). **d**. Normal tissue.Lamina propria. Scattered positive nuclei of mononuclear cells in the lamina propria of normal mucosa (arrow heads). **e**. UC tissue. Purulent material from ulcer. Negative neutrophils in purulent material (arrow heads) positive nuclei of mononuclear cells (arrows).

For the inducible HSP70, we found weak focal cytoplasmic positivity of the surface epithelium, and mild focal nuclear staining at the base of some crypts. There was nuclear staining of some MC in the lamina propria (Figure 2a, c, d).

### Immunohistochemistry - UC

For the constitutive HSP70, we observed a tendency for downregulation in both surface epithelium and crypts. However, staining was increased in the lamina propria, mainly MC, and to a lesser degree in the PMN cells (Figure 1b, d, f).

For the inducible HSP70, a few of the mild and moderate activity epithelial specimens showed weak staining on the surface; the severe activity specimens showed almost no staining. The crypts showed even fewer cases of weak staining compared to the surface epithelium. In the lamina propria, the staining of PMN cells was increased in the biopsies from 22 patients regardless of the severity of the biopsy findings (Table 1) (Figure 2b). In the biopsies of the rest 7 patients, negative PMN were observed (2e).

**Table 1:** Expression* of constitutive and inducible HSP70 in tissue resection

	**Controls N = 12**	Degree of expression* N = 57		
	**Controls N = 12**	**UC = Mild N = 24**	**UC = Moderate N = 27**	**UC = Severe N = 6**
*HSP70 constitutive*				
Surface epithelium				
Nucleus	++ (9/12)	++ (13/24)	++ (16/27)	++ (3/6)
	+ (1/12)	+ (2/24)	+ (5/27)	+ (1/6)
	0– + (1/12)	0 - + (6/24)	0 - + (5/27)	0 - + (2/6)
		0 (3/24)	0 (1/27)	
Cytoplasm	++ (8/12)	++ (7/24)	++ (5/27)	++ (3/6)
	+ (2/12)	+ (7/24)	+ (12/27)	+ (0/6)
	0– + (2/12)	0 - + (6/24)	0 - + (8/27)	0 - + (2/6)
		0 (4/24)	0 (2/27)	0 (1/6)
Crypts				
Nucleus	++ (7/12)	++ (2/24)	++ (13/27)	++ (3/6)
	+ (2/12)	+ (9/24)	+ (5/27)	+ (1/6)
	0– + (3/12)	0 - + (9/24)	0 - + (8/27)	0 - + (2/6)
		0 (4/24)	0 (1/27)	
Cytoplasm	++ (7/12)	++ (3/24)	++ (6/27)	++ (3/6)
	0– + (4/12)	+ (8/24)	+ (11/27)	+ (0/6)
	0 (1/12)	0 - + (9/24)	0 - + (7/27)	0 - + (1/6)
		0 (4/24)	0 (3/27)	0 (2/6)
Lamina propria				
Mononuclear cells	+ (6/12)	+ (14/24)	+ (18/27)	+ (5/6)
	0 (6/12)	0 (10/24)	0 (9/27)	0 (1/6)
Polymorphonuclear cells	0 (12/12)	+ (7/24)	+(16/27)	+ (4/6)
		0 (17/24)	0 (11/27)	0 (2/6)
*HSP70 inducible*				
Surface epithelium				
Nucleus	0– + (2/12)	0 - + (4/24)	0 - +(4/27)	———————
	0 (10/12)	0 (20/24)	0 (23/27)	0 (6/6)
Cytoplasm	0– + (4/12)	0 - + (5/24)	0 - +(6/27)	0 - + (2/6)
	0 (8/12)	0 (19/24)	0 (21/27)	0 (4/6)
Crypts				
Nucleus	0– + (2/12)	0 - + (2/24)	0 - + (2/27)	———————-
	0 (10/12))	0 (22/24)	0 (25/27)	0 (6/6)
Cytoplasm	0– + (2/12)	———————	0 - +(1/27)	———————
	0 (10/12)	0 (24/24)	0 (26/27)	0 (6/6)
Lamina propria				
Mononuclear cells	+ (1/12)	+ (1/24)	+ (1/27)	———————
	0 (11/12)	0 (23/24)	0 (26/27)	0 (6/6)
Polymorphonuclear cells	0 (12/12)	+ (16/24)	+ (21/27)	+ (5/6)
		0 (8/24)	0 (6/27)	0 (1/6)

*Staining: no staining, 0; weak, 0– +; medium, +; intense, ++.

### Psychometric tools

Most scores for all psychometric tools were normal (Table 2). Except for the hospital anxiety scale, scores for all psychometric tools were higher in patients with positive immunoreactivity in the PMN cells of the inducible HSP70 (Table 3). The psychometric scales were selected in order to increase the reliability and validity of the results on depression and anxiety. As can be seen in Table 4, the psychometric scales correlate significantly to eachother despite the lack of correlation with the duration of the disease (in years).

**Table 2:** **Distribution of patients in normal, a significant case**^**b**^**, or clinical case**^**c**^**, by scores obtained in psychometric scales**

	**Normal**^**a**^**N (%)**	**Significant case**^**b**^**N (%)**	**Clinical Case**^**c**^**N (%)**
State anxiety	17 (58.6)	7 (24.1)	5 (17.2)
Trait anxiety	14 (48.3)	12 (41.4)	3 (10.3)
ZDRS	28 (96.6)	-	1 (3.4)
Hospital anxiety	19 (65.5)	5 (17.2)	5 (17.2)
Hospital depression	21 (72.4)	6 (20.7)	2 (6.9)

*Abbreviations*: *ZDRS* Zung depression rating scale.

a:Normal: State anxiety 20–39; Trait anxiety 20–39; ZDRS 20–50; Hospital anxiety 0–7; Hospital depression 0–7.

b:Significant case: State anxiety 40–54; Trait anxiety 40–54; ZDRS not applicable; Hospital anxiety 8–11; Hospital depression 8–11.

c:Clinical case: State anxiety >54; Trait anxiety >54; ZDRS > 50; Hospital anxiety >11; Hospital depression ≥11.

**Table 3:** Mean scores for psychometric tools, in the total population, and immunoreactivity of inducible HSP70

	**Total mean (SD) N = 29**	**HSP70i PMN positive mean (SD) N = 22**	**HSP70i PMN negative mean (SD) N = 7**	**P value (t-test)**	**P value (Mann–Whitney)**	**P value (corrected for corticosteroids)**
State anxiety	40.5 (13.2)	43.5 (13.8)	31.1 (4.3)	0.001	0.028	0.034
Trait anxiety	39.4 (10.0)	42.6 (9.1)	29.9 (5.4)	0.002	0.001	0.001
ZDRS	37.0 (6.8)	38.7 (6.2)	30.0 (4.5)	0.007	0.006	0.002
Hospital anxiety	6.2 (3.9)	7.0 (3.8)	3.7 (3.4)	0.056	0.048	0.070
Hospital depression	5.1 (3.6)	6.0 (3.6)	2.4 (1.6)	0.019	0.018	0.019
CAI	6.4 (3.1)	7.1 (3.1)	4.1 (2.0)	0.026	0.028	0.033
		5.5 (2.5)	3.4 (1.8)			

*Abbreviations*: *ZDRS* Zung-depression-rating-scale.

**Table 4:** Correlations between psychometric scales and between duration of disease (in years) and psychometric scales

			**Duration of disease**	**STAI1**	**STAI2**	**ZUNGDEPRESSION**	**HA**	**HD**
Spearman’s rho	Duration of Disease	Correlation Coefficient	1,000	-,031	-,084	-,127	,020	-,059
		Sig. (2-tailed)	.	,884	,691	,544	,926	,780
		N	25	25	25	25	25	25
	STAI1	Correlation Coefficient	-,031	1,000	,652**	,503**	,508**	,447*
		Sig. (2-tailed)	,884	.	,000	,005	,005	,015
		N	25	29	29	29	29	29
	STAI2	Correlation Coefficient	-,084	,652**	1,000	,678**	,618**	,607**
		Sig. (2-tailed)	,691	,000	.	,000	,000	,000
		N	25	29	29	29	29	29
	ZUNGDEPRESSION	Correlation Coefficient	-,127	,503**	,678**	1,000	,334	,512**
		Sig. (2-tailed)	,544	,005	,000	.	,076	,005
		N	25	29	29	29	29	29
	HA	Correlation Coefficient	,020	,508**	,618**	,334	1,000	,671**
		Sig. (2-tailed)	,926	,005	,000	,076	.	,000
		N	25	29	29	29	29	29
	HD	Correlation Coefficient	-,059	,447*	,607**	,512**	,671**	1,000
		Sig. (2-tailed)	,780	,015	,000	,005	,000	.
		N	25	29	29	29	29	29

*Correlation is significant at the 0.05 level (2-tailed).

**Correlation is significant at the 0.01 level (2-tailed).

The expression of the inducible HSP70 was similar in patients with and without corticosteroids: 70% positive immunoreactivity for the inducible HSP70 in those receiving corticosteroids and 79% in those not receiving corticosteroids (P = 0.593).

Logistic regression showed a strong positive relationship between immunoreactivity of the inducible HSP70 in the PMN cells and scores in the trait anxiety, ZDRS, and hospital depression scales, (Odds ratios 1.3, 1.3, and 1.5; P = 0.018, 0.023, and 0.038; Wald test, 5.6, 5.2, and 4.3 respectively). The same relations were found after controlling for corticosteroids administration (Odds ratio 1.3, 1.4, 1.5; P, 0.021, 0.019, and 0.034; Wald test, 5.3, 5.5, and 4.5, respectively). Moreover, a weaker but significant correlation was established between the inducible HSP70 in PMN cells and the CAI (Odds ratio 1.654; P = 0.049; Wald test 3.858) and similarly after controlling for corticosteroids administration (Odds ratio 1.649; P = 0.051; Wald test = 1.649) (Table 3). Duration of the disease did not correlate with the psychometric scores (Table 4) and did not correlate with the HSP70 induction in the PMN cells either (results not shown). Also, disease activity did not correlate with HSP70 induction in PMN cells. In the crosstabulation of disease activity and HSP70 induction in PMN cells (Table 5), we observed that the percentage of present/absent proteins increases/decreases according to disease activity. The statistical significance was examined by the chi-square test and the results (Chi-square = 2,77; df = 2; Asymp.Sig (two-sided) p = 0,2503) showed that there was no statistical significance between HSP70 induction in MN cells and disease activity.

**Table 5:** Disease activity and HSP70i PMN crosstabulation

			**HSP70i PMN**		**Total**
			**Absent**	**Present**	
DISEASE ACTIVITY	MILD	Count	3	3	6
		%	50,0%	50,0%	100,0%
	MEDIUM	Count	3	14	17
		%	17,65%	82,35%	100,0%
	SEVERE	Count	1	5	6
		%	16,7%	83,3%	100,0%
Total		Count	7	22	29
		%	24,1%	75,9%	100,0%

## Discussion

This study investigated the relationship between HSP70 induction in the surface epithelium, crypts, MC, and PMN cells of colonic mucosa with levels of anxiety and depression in patients with UC. It is the first published study to show a positive psychological correlation between the induction of the cytoprotective, antiapoptotic HSP70 in PMN cells that are known to perpetuate inflammation in UC patients.

**Figure 3:**
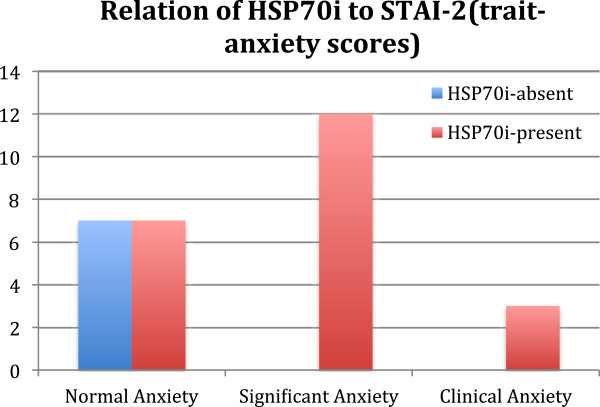
**The relationship between the degrees of anxiety as a trait (STAI2) and the presence or absence of HSP70 immunoreactivity in PMN cells.** The number of patients is depicted on the vertical axis, whereas the severity of anxiety –as expressed through the State Trait Anxiety Inventory II (as a trait) is depicted on the horizontal axis.

As is already known, UC patients seem to have increased sympathetic autonomic activity compared to controls, and the severity of their symptoms is associated with measures of personality-related anxiety, but not situational anxiety [10]. It is of interest to note that our statistical evaluation showed that anxiety as a personality trait had a stronger association than situational anxiety to HSP70 induction in PMN cells (Figure 3).

Histopathology revealed that PMN cells showed clear expression of HSP70i in the biopsies of UC patients. We also found sparse and weak HSP70 induction in the surface epithelium and crypts. Although we used the same antibody, our findings did not confirm previous observations of clear mucosal and submucosal staining of epithelial and endothelial cells (where some immunoreactivity has already been described for PMN as well) [22]. Our results, however, support evidence of HSP70i downregulation in the biopsies of UC patients rendering their mucosa vulnerable to inflammation-induced injury [19]. This observation supports the tenet that HSP cytoprotection may eventually become exhausted in any chronic disease which causes massive protein misfolding and aggregation [16]. Additionally, the even more rare expression of HSP70i that we observed in the crypts of UC specimens compared to the surface epithelium agrees with previous results [19]. HSP70i becomes downregulated in the epithelium and crypts, confirming previous research and rendering the intestine more vulnerable to inflammation. Moreover, HSP70 becomes induced in the PMN cells (and not the epithelium) of most patients with active disease. PMN cells are implicated in the autoimmune mechanism of mucosal destruction in UC through the release of ROS.Through the induction of HSP70i, PMN cells become more resistant to apoptosis and cell death. The induction of HSP70i in PMN cells correlates with anxiety and depression scores (and not with the downregulated HSP70i of the epithelium).

Current research on IBD treatment is focusing on the inhibition of PMN transmigration across mucosal epithelia and on novel therapies that promote PMN apoptosis [36-39]. HSP70 induction, which protects PMN cells from apoptosis and at the same time significantly correlates with the degree of anxiety and depression of UC patients, might be a bringing point for the role that psychological factors play in the natural history of the disease, as expressed by UC patients and observed in relevant studies [4,6].

It would be of great clinical interest to clarify whether the alleviation of anxiety and depression symptoms decreases HSPi in PMN cells of the colon mucosa. The increase of attenuation of HSP70 induction and its anti-apoptotic effect on PMN relating to anxiety and depression could serve as a useful biological marker for the in-depth study of auto-immune and psychological interventions in UC.

The cross-sectional design of our study does not allow us to infer causality direction. The small sample size is a limitation that we tried to counterbalance by the statistical methods chosen. The homogeneity of the samples, however, strengthens the results, as there is evidence that the use of mixed samples from patients with UC and CD is a persistent methodological flaw in human studies on the impact of psychological factors on the course of the IBD. This is because of the differences in the nature of the two diseases. Also, prospective studies with mixed samples have been almost entirely negative [23,40]. Moreover, we did not compare the patients with active disease (where we would observe HSP70 induction) with the patients with quiescent disease, which could provide us with more data.

## Conclusion

In conclusion, our preliminary results have shown that further studies on the psychosomatic nature of IBD are warranted and should focus on the use of HSP70 as a biological marker.

## Competing interests

The authors declare that they have no competing interests.

## Authors’ contributions

IV carried out the psychometric interviews, collected the biopsies from the gastroenterologist, did the statistical analysis and wrote the manuscript draft. CB has diagnosed all biopsies and interpreted all data. MT performed all immunohistochemical staining. LAA collected specimens and data and participated with CB in diagnosis and interpretation of biopsies. CG performed the colonoscopies and provided the histological material. MGC provided the psychometric questionnaires and participated in the interpretation of psychometric results after the statistical analysis. ME contributed to the statistical analysis of the psychometric questionnaires and the interpretation of data. GNP supervised the administration and collection of the psychometric questionnaires and provided literature concerning the psychological aspects of ulcerative colitis. EP supervised the diagnosis and interpretation of all histopathological material. PNS conceived the study and has supervised the histopathology and contributed to the synthesis of the manuscript. All authors read and approved the final manuscript.

## Pre-publication history

The pre-publication history for this paper can be accessed here:

http://www.biomedcentral.com/1471-230X/14/77/prepub

## References

[B1] Sartor RB Mechanisms of disease: pathogenesis of Crohn’s disease and ulcerative colitis Nat Clin Pract Gastroenterol Hepatol 2006 3 7 390 407 10.1038/ncpgasthep0528 16819502

[B2] Maunder RG Levenstein S The role of stress in the development and clinical course of inflammatory bowel disease: epidemiological evidence Curr Mol Med 2008 8 4 247 252 10.2174/156652408784533832 18537632

[B3] Mawdsley JE Macey MG Feakins RM Langmead L Rampton DS The effect of acute psychologic stress on systemic and rectal mucosal measures of inflammation in ulcerative colitis Gastroenterology 2006 131 2 410 419 10.1053/j.gastro.2006.05.017 16890594

[B4] Mikocka-Walus AA Turnbull DA Moulding NT Wilson IG Andrews JM Holtmann GJ Controversies surrounding the comorbidity of depression and anxiety in inflammatory bowel disease patients: a literature review Inflamm Bowel Dis 2007 13 2 225 234 10.1002/ibd.20062 17206706

[B5] Hauser W Janke KH Klump B Hinz A Anxiety and depression in patients with inflammatory bowel disease: comparisons with chronic liver disease patients and the general population Inflamm Bowel Dis 2011 17 2 621 632 10.1002/ibd.21346 20848528

[B6] Tache Y Bernstein CN Evidence for the role of the brain-gut axis in inflammatory bowel disease: depression as cause and effect? Gastroenterology 2009 136 7 2058 2061 10.1053/j.gastro.2009.04.032 19406133 PMC3675266

[B7] Ghia JE Blennerhassett P Deng Y Verdu EF Khan WI Collins SM Reactivation of inflammatory bowel disease in a mouse model of depression Gastroenterology 2009 136 7 2280 2288 10.1053/j.gastro.2009.02.069 19272381

[B8] Kubera M Obuchowicz E Goehler L Brzeszcz J Maes M In animal models, psychosocial stress-induced (neuro)inflammation, apoptosis and reduced neurogenesis are associated to the onset of depression Prog Neuropsychopharmacol Biol Psychiatry 2011 35 744 759 10.1016/j.pnpbp.2010.08.026 20828592

[B9] Srinath AI Walter C Newara MC Szigethy EM Pain management in patients with inflammatory bowel disease: insights for the clinician Therap Adv Gastroenterol 2012 5 5 339 357 10.1177/1756283X12446158 22973418 PMC3437534

[B10] Ganguli SC Kamath MV Redmond K Chen Y Irvine EJ Collins SM Tougas G A comparison of autonomic function in patients with inflammatory bowel disease and in healthy controls Neurogastroenterol Motil 2007 19 12 961 967 17931336 10.1111/j.1365-2982.2007.00987.x

[B11] Rachmilewitz D Coated mesalazine (5-aminosalicylic acid) versus sulphasalazine in the treatment of active ulcerative colitis: a randomised trial BMJ 1989 298 66 82 86 2563951 10.1136/bmj.298.6666.82 PMC1835436

[B12] Matsuo K Zhang X Ono Y Nagatomi R Acute stress-induced colonic tissue HSP70 expression requires commensal bacterial components and intrinsic glucocorticoid Brain Behav Immun 2009 23 1 108 115 10.1016/j.bbi.2008.07.013 18760344

[B13] Wu T Tanguay RM Antibodies against heat shock proteins in environmental stresses and diseases: friend or foe? Cell Stress Chaperones 2006 11 1 1 12 10.1379/CSC-155R.1 16572724 PMC1400608

[B14] Hayase T Yamamoto Y Yamamoto K Muso E Shiota K Hayashi T Similar effects of cocaine and immobilization stress on the levels of heat shock proteins and stress-activated protein kinases in the rat hippocampus and on swimming behaviors: the contribution of dopamine and benzodiazepine receptors Beh Pharmacol 2003 14 7 551 562 10.1097/00008877-200311000-00008 14557723

[B15] Srivastava P Jobs for ancient chaperones Sci Am 2008 299 1 32 37 10.1038/scientificamerican0708-50 18623964

[B16] Sreedhar AS Csermely P Heat shock proteins in the regulation of apoptosis: new strategies in tumor therapy: a comprehensive review Pharmacol Ther 2004 101 3 227 257 10.1016/j.pharmthera.2003.11.004 15031001

[B17] Dronjak S Gavrilovic L Filipovic D Radojcić MB Immobilization and cold stress affect sympatho-adrenomedullary system and pituitary-adrenocortical axis of rats exposed to long-term isolation and crowding Physiol Behav 2004 81 3 409 415 10.1016/j.physbeh.2004.01.011 15135012

[B18] Fleshner M Campisi J Amiri L Diamond DM Cat exposure induces both intra- and extracellular Hsp72: the role of adrenal hormones Psychoneuroendocrinology 2004 29 9 1142 1152 10.1016/j.psyneuen.2004.01.007 15219638

[B19] Hu S Ciancio MJ Lahav M Fujiya M Lichtenstein L Anant S Musch MW Chang EB Translational inhibition of colonic epithelial heat shock proteins by IFN-gamma and TNF-alpha in intestinal inflammation Gastroenterology 2007 133 6 1893 1904 10.1053/j.gastro.2007.09.026 18054561 PMC2180161

[B20] Liu TS Musch MW Sugi K Sugi K Walsh-Reitz MM Ropeleski MJ Hendrickson BA Pothoulakis C Lamont JT Chang EB Protective role of HSP72 against clostridium difficile toxin a-induced intestinal epithelial cell dysfunction Am J Physiol Cell Physiol 2003 284 4 C1073 C1082 10.1152/ajpcell.00134.2002 12490434

[B21] Tao Y Hart J Lichtenstein L Joseph LJ Ciancio MJ Hu S Chang EB Bissonnette M Inducible heat shock protein 70 prevents multifocal flat dysplastic lesions and invasive tumors in an inflammatory model of colon cancer Carcinogenesis 2009 30 1 175 182 19005184 10.1093/carcin/bgn256 PMC2722142

[B22] Ludwig D Stahl M Ibrahim ET Wenzel BE Drabicki D Wecke A Fellermann K Stange EF Enhanced intestinal expression of heat shock protein 70 in patients with inflammatory bowel diseases Dig Dis Sci 1999 44 7 1440 1447 10.1023/A:1026616221950 10489932

[B23] Maunder RG Evidence that stress contributes to inflammatory bowel disease: evaluation, synthesis, and future directions Inflamm Bowel Dis 2005 11 6 600 608 10.1097/01.MIB.0000161919.42878.a0 15905709

[B24] Lochart-Mummery HE Morson BC Crohn’s disease (regional enteritis) of the large intestine and its distinction from ulcerative colitis Gut 1960 1 87 105 10.1136/gut.1.2.87 14417801 PMC1413217

[B25] Liakos A Giannitsi S Reliability and validity of teh greek translation of the Spielberger’s anxiety inventory Engefalos 1984 21 71 76

[B26] Spielberger DD Gorsuch RL Lushene RE Manual for the state-trait anxiety inventory 1970 Palo Alto: Consulting Psychologists Press

[B27] Kvaal K Ulstein I Nordhus IH Engedal K The spielberger state-trait anxiety inventory (STAI): the state scale in detecting mental disorders in geriatric patients Int J Geriatr Psychiatry 2005 20 7 629 634 10.1002/gps.1330 16021666

[B28] Fountoulakis K Iacovides A Kleanthous S Samolis S Kaprinis SG Sitzoglou K St Kaprinis G Bech P Reliability, validity and psychometric properties of the greek translation of the center for epidemiological studies-depression (CES-D) scale BMC Psychiatry 2001 1 3 10.1186/1471-244X-1-3 11454239 PMC34551

[B29] Zung WW Richards CB Short MJ Self-rating depression scale in an outpatient clinic Further validation of the SDS. Arch Gen Psychiatry 1965 13 6 508 515 10.1001/archpsyc.1965.01730060026004 4378854

[B30] Michopoulos I Douzenis A Kalkavoura C Christodoulou C Michalopoulou P Kalemi G Fineti K Patapis P Protopapas K Lykouras L Hospital anxiety and depression scale (HADS): validation in a greek general hospital sample Ann Gen Psychiatry 2008 7 4 10.1186/1744-859X-7-4 18325093 PMC2276214

[B31] Zigmond AS Snaith RP The hospital anxiety and depression scale Acta Psychiatr Scand 1983 67 6 361 370 10.1111/j.1600-0447.1983.tb09716.x 6880820

[B32] Porcelli P Leoci C Guerra V A prospective study of the relationship between disease activity and psychologic distress in patients with inflammatory bowel disease Scand J Gastroenterol 1996 31 8 792 796 10.3109/00365529609010354 8858749

[B33] Dieleman LA Palmen MJ Akol H Bloemena E Peña AS Meuwissen SG Van Rees EP Chronic experimental colitis induced by dextran sulphate sodium (DSS) is characterized by Th1 and Th2 cytokines Clin Exp Immunol 1998 114 3 385 391 10.1046/j.1365-2249.1998.00728.x 9844047 PMC1905133

[B34] Hanauer SB Robinson M Pruitt R Lazenby AJ Persson T Nilsson LG Walton-Bowen K Haskell LP Levine JG Budesonide enema for the treatment of active, distal ulcerative colitis and proctitis: a dose-ranging study. U.S. Budesonide enema study group Gastroenterology 1998 115 525 532 10.1016/S0016-5085(98)70131-3 9721148

[B35] Malusecka E Zborek A Krzyzowska-Gruca S Krawczyk Z Immunohistochemical detection of the inducible heat shock protein hsp70: a methodological study J Histochem Cytochem 2006 54 2 183 190 10.1369/jhc.5A6748.2005 16204226

[B36] Chin AC Parkos CA Neutrophil transepithelial migration and epithelial barrier function in IBD: potential targets for inhibiting neutrophil trafficking Ann N Y Acad Sci 2006 1072 276 287 10.1196/annals.1326.018 17057207

[B37] Lindberg A Eberhardson M Karlsson M Karlén P Long-term follow-up with granulocyte and monocyte apheresis re-treatment in patients with chronically active inflammatory bowel disease BMC Gastroenterol 2010 10 73 10.1186/1471-230X-10-73 20604939 PMC2914086

[B38] Miehsler W Weichselberger M Offerlbauer-Ernst A Dejaco C Reinisch W Vogelsang H Machold K Stamm T Gangl A Moser G Which patients with IBD need psychological interventions? A controlled study Inflamm Bowel Dis 2008 14 9 1273 1280 10.1002/ibd.20462 18393373

[B39] Mollinedo F Gajate C Morales AI del Canto-Jañez E Justies N Collía F Rivas JV Modolell M Iglesias A Novel anti-inflammatory action of edelfosine lacking toxicity with protective effect in experimental colitis J Pharmacol Exp Ther 2009 329 2 439 449 10.1124/jpet.108.148254 19244550

[B40] Bailey L Vardulaki K Langham J Chandramohan D Introduction to epidemiology 2005 London: Open University Press

